# Can Ecological Niche Modeling of Prickly Juniper (*Juniperus oxycedrus* L.) Predict Future Forest Distribution Limits in Central Anatolia?

**DOI:** 10.3390/plants15050743

**Published:** 2026-02-28

**Authors:** Derya Gülçin, Javier Velázquez, Gamze Tuttu, Daniel Sánchez-Mata, Ebru Ersoy Tonyaloğlu, Kerim Çiçek, Sezgin Ayan, Mehmet Sezgin, Ahmet Varlı, Ali Uğur Özcan

**Affiliations:** 1 Department of Landscape Architecture, Faculty of Agriculture, Aydın Adnan Menderes University, Aydın 09100, Türkiye; derya.yazgi@adu.edu.tr (D.G.); ebru.ersoy@adu.edu.tr (E.E.T.); 2 TEMSUS Research Group, Catholic University of Ávila—Universidad Católica de Ávila (UCAV), 05005 Ávila, Spain; javier.velazquez@ucavila.es (J.V.); kerim.cicek@ege.edu.tr (K.Ç.); auozcan@karatekin.edu.tr (A.U.Ö.); 3 Department of Environment and Agroforestry, Faculty of Sciences and Arts, Catholic University of Ávila, 05005 Ávila, Spain; 4 Department of Forest Engineering, Faculty of Forestry, Çankırı Karatekin University, Çankırı 18200, Türkiye; gamzeertugrul@karatekin.edu.tr; 5 Botany Unit, Faculty of Pharmacy, Complutense University of Madrid, 28040 Madrid, Spain; 6 Department of Organismic and Evolutionary Biology (OEB), Harvard University Herbaria, Harvard University, Cambridge, MA 02138, USA; 7 Section of Zoology, Department of Biology, Faculty of Science, Ege University, Izmir 35040, Türkiye; 8 Department of Silviculture, Faculty of Forestry, Kastamonu University, Kastamonu 37150, Türkiye; sezginayan@kastamonu.edu.tr; 9 Department of Biology, Faculty of Science, Çankırı Karatekin University, Çankırı 18200, Türkiye; sezgin@karatekin.edu.tr; 10 Aegean Agricultural Research Institute, Republic of Türkiye Ministry of Agriculture and Forestry, Izmir 35040, Türkiye; ahmetvarli@ogm.gov.tr; 11 Department of Landscape Architecture, Faculty of Forestry, Çankırı Karatekin University, Çankırı 18200, Türkiye

**Keywords:** prickly juniper, ecological niche modeling, climate change, habitat suitability, MaxEnt

## Abstract

Climate change is expected to alter the distribution limits of woody species in Mediterranean and semi-arid regions, especially near forest–steppe transition zones. In this study, ecological niche modeling (ENM) was applied to examine the current and future habitat suitability of prickly juniper (*Juniperus oxycedrus* L.) in Türkiye under three Shared Socioeconomic Pathways (SSP1-2.6, SSP3-7.0, and SSP5-8.5) for the periods 2011–2040, 2041–2070, and 2071–2100. Species–environment relationships were quantified using the Maximum Entropy (MaxEnt) algorithm. From 48 candidate MaxEnt models, the optimal model was selected based on statistical performance and showed a high mean training AUC (AUC = 0.869, SD = 0.017). Null model testing confirmed that predictive performance exceeded random expectations (AUC_null_ = 0.593, SD = 0.011; Z = 28.294, *p* < 0.00001). Among all predictors, precipitation of the driest month (bio14) and slope showed the highest contributions, accounting for 24.9% and 24.3%, respectively. Present-day suitability reveals that *J. oxycedrus* has a wide distribution in the interior Anatolian and Mediterranean uplands. Future projections indicate limited habitat loss during the early projection period, followed by substantial reductions toward the end of the century, particularly under high-emission scenarios. Late-century projections suggest that suitable habitats become increasingly restricted to mountainous areas, including the Taurus range and selected highland regions of Central and northern Türkiye. Overall, the findings underline that climate adaptation is closely linked to how biome boundaries are managed in relation to ecological thresholds. Expanding forest cover beyond natural environmental limits may not represent an effective adaptation strategy.

## 1. Introduction

Prior to large-scale degradation, Central Anatolia was largely covered by forests, except for the saline Konya Closed Basin, the Tuz Lake Basin, and the gypsum-rich areas of Çankırı [1,2,3,4]. This spatial configuration indicates the widespread presence of ecologically suitable niches for tree species under climatic and edaphic conditions of the region [5,6]. Roberts et al. [7] reported that the gradual increase in arboreal pollen during the Early Holocene came to an end around 7000–6500 calibrated years BP. This shift indicates a substantial disruption in forest cover, despite the persistence of climatic suitability. Central Anatolia, one of the earliest regions of sustained human settlement, has undergone substantial environmental transformations associated with long-term human activity [8,9]. Continuous human presence expanded the gap between the potential distribution of forests and their actual extent [10]. Archeological excavations and pollen records document extensive forest loss and major structural change across the region [11,12,13]. Wright et al. [14] described forest change during the Bronze and Iron Ages as a distinct anthropological signature linked to the environmental influence of the Hittite State. Regional archeological studies show strong consistency in the timing of forest degradation across sites [15,16,17]. This temporal correspondence indicates that human pressure emerged as the dominant regional force, exceeding the constraints imposed by ecological suitability. Palynological records also support this interpretation [7,18,19]. On this basis, it can be inferred that the environmental niches governing the potential distribution of tree species in Central Anatolia shifted progressively under sustained human pressure [20].

Central Anatolia lies at the transition between the Irano-Turanian and Mediterranean floristic regions and has a complex biogeographical setting [3]. The Central Anatolian plateau has marked thermal contrasts. Precipitation is irregular and generally low, with annual means close to 200 mm, and drought-related water deficits prevail across the region [21]. According to the Thornthwaite climate classification, this region is described as semi-arid to semi-humid, with moisture availability near levels that limit vegetation distribution [22]. Across the Mediterranean Basin, global warming results in higher temperatures, reduced water availability, and increased drought frequency [23]. Climate projections for Central Anatolia indicate an increase in high-intensity precipitation concurrent with a decline in mean annual rainfall, a combination that undermines hydrological balance [24]. Based on high-resolution ERA-Interim and HadGEM2-CC model outputs, Yılmaz [25] projected that semi-arid zones in the region will expand to nearly twice their present extent under the Holdridge life-zone humidity classification for both current and future climatic conditions in Türkiye.

Due to its closed-basin position and proximity to desert biomes, Central Anatolia hosts ecosystems that are highly sensitive to global warming [8]. At present, the main tree species forming the forest structure of these ecosystems are Anatolian black pine (*Pinus nigra* subsp. *pallasiana* var. *caramanica*), junipers (*Juniperus* spp.), and oaks (*Quercus* spp.). These forests decrease in number as one moves from the large and small remnant forest blocks surrounding the region toward the central areas [26]. Tree height, individual density, and canopy cover decline markedly toward Central Anatolia [27]. Climate projections indicate that the region is expected to have a reduced potential forest area in the future compared to the present. Model outputs show that the potential distribution areas of the region’s main forest components—*P. nigra* [28], *J. excels* [26], and *Q. vulcanica* [29]—are projected to shrink. Arslan and Örücü [30] indicate that those projections for 2050 and 2070 point to substantial contractions in the potential distribution of *P. nigra*. In line with these projections, Yıldız et al. [8] reported that, despite extensive afforestation efforts, nearly two-thirds of black pine seedlings did not survive beyond the first 8–10 years at afforestation sites of different ages in the Tuz Gölü within the Konya Basin. In contrast, the species-rich semi-natural steppe ecosystems of the Irano-Turanian floristic region extending into Eastern and Central Anatolia are extremely important due to the wide range of essential ecosystem services they provide, including biodiversity conservation, economic value, and nutrient cycling. Central Anatolia lies within the boundaries of the Irano-Anatolian biodiversity hotspot, identified by Mittermeier et al. [31] as one of the seven important grassland regions in the Palearctic realm [32]. However, these steppe areas lack adequate national-level conservation targets [32]. In large parts of Central Anatolia, present-day vegetation occupies landscapes that once supported forests; however, it shows levels of degradation comparable to forest ecosystems and has largely transitioned into a secondary steppe state [3]. At the same time, Tavşanoğlu and Bernardi [33] emphasize that steppes have long coexisted with forests and should not be viewed solely as degraded derivatives, but rather as alternative biome states within forest-dominated systems. Although research on steppe and forest–steppe mosaics has expanded in recent years [5], knowledge of the compositional and structural characteristics of these transitional landscapes remains limited [34].

At present, forest and steppe ecosystems in Central Anatolia form a mosaic landscape. However, it remains uncertain whether this configuration will endure under increasing human pressure and climate change. Therefore, identifying the future potential boundaries of these two major ecosystems is of critical importance. Accordingly, the main objective of this study was to assess the current and future habitat suitability of prickly juniper (*Juniperus oxycedrus* L.) and to evaluate its potential role in indicating future forest distribution limits in Central Anatolia under climate change. This study focuses on the forest–steppe boundary zone for four main reasons: (i) to enhance the climate change adaptation capacity of steppe ecosystems by strengthening them instead of directly implementing afforestation policies in non-forested areas; (ii) to prioritize the conservation of existing remnant forest areas; (iii) to reduce time and resource losses by identifying potential forest ecosystem areas in advance rather than relying on trial-and-error practices; and (iv) to generate spatial information to mitigate the barrier effect of large treeless areas that may impede the northward and upward altitudinal migration of species under climate change. Within this scope, the potential distribution of the target species was modeled using ecological niche modeling under current and future climate scenarios. The information presented here can support forestry planning by identifying areas where future climatic conditions are expected to remain suitable for forests. It may provide guidance on conservation priorities while simultaneously avoiding afforestation in ecologically unsuitable areas.

## 2. Results

### 2.1. Model Performance

Among the 48 candidate models generated using the maximum entropy approach, the optimal model was selected based on a balance between statistical accuracy and simplicity. Model selection was evaluated through a null model test. The selected model included linear, quadratic, hinge, and product features, with a regularization parameter of 0.5, and yielded a high mean training AUC value (AUC = 0.869, SD = 0.017). A comparison between the empirical model and the null model demonstrated a statistically significant difference in predictive performance (AUC_null_ = 0.593, SD = 0.011, Z = 28.294, *p* < 0.00001; Appendix A, Table A1). This result confirms that the selected model performed well beyond random expectations (Appendix A, Table A2).

### 2.2. Present-Day and Future Projections

**Figure A1:**
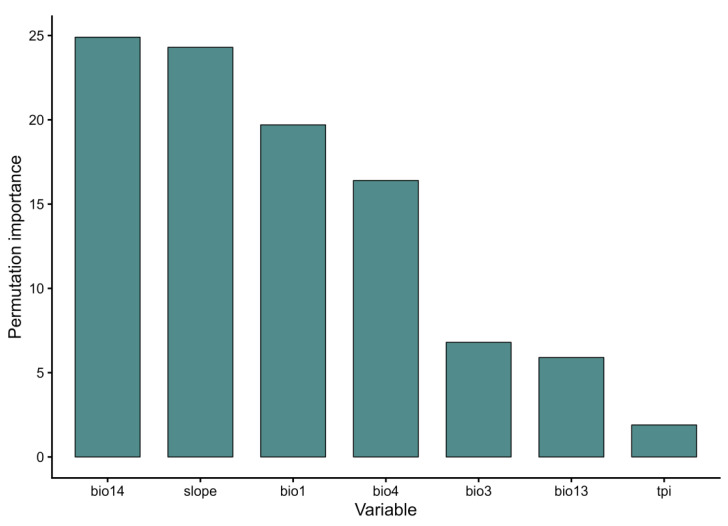
Relative importance of environmental variables in the ecological niche model for the prickly juniper (*Juniperus oxycedrus* L.) based on permutation importance.

Habitat suitability of *J. oxycedrus* in Türkiye arises from the influence of climatic and topographic variables, which demonstrates this species’ sensitivity to broad-scale climate patterns and local environmental conditions. Among all predictors, precipitation of the driest month (bio14) showed the highest contribution (24.9%), which indicates the important role of summer drought stress in determining suitable habitats (Appendix A, Figure A1). Slope accounted for the second-highest contribution (24.3%). Terrain with a marked slope generally has lower land-use intensity and more stable soil and drainage conditions. Temperature-related variables also exerted a strong influence. Mean annual temperature (bio1; 19.7%) and temperature seasonality (bio4; 16.4%) indicate that this species inhabits areas with moderate thermal conditions and shows limited tolerance to extreme cold or heat. Isothermality (bio3) contributed at an intermediate level (6.8%), which suggests sensitivity to short-term temperature variations relative to annual patterns. By comparison, precipitation of the wettest month (bio13) showed a relatively low contribution (5.9%). The topographic position index (TPI) showed the lowest contribution (1.9%), yet it still indicated distinctions between more exposed and less exposed sites.

**Figure 1:**
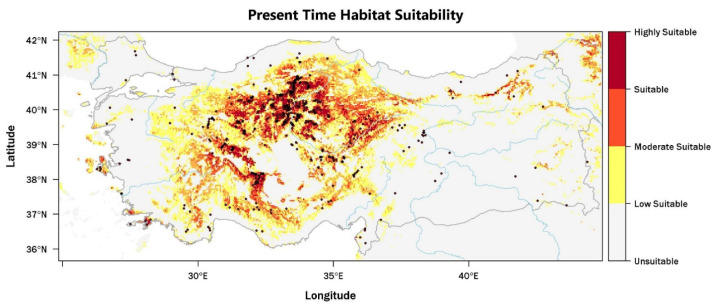
The extent of the study area projecting present-day potential distribution and recorded occurrences of the prickly juniper (*Juniperus oxycedrus* L.) under future climate scenarios in Türkiye. Black dots represent occurrence records. The probability of occurrence ranges from 0 (gray, lowest probability) to 1 (black, highest probability).

The present-day habitat suitability map integrates known occurrence records with model-based predictions across the range of species found in Türkiye (Figure 1). Areas of high and moderate suitability occur predominantly across Central Anatolia, the interior Mediterranean region, and the transitional belt between the Central Anatolian Plateau and the Black Sea region. These areas are mainly associated with mountainous and elevated terrain that exhibits considerable microhabitat heterogeneity. Observed occurrences show close spatial correspondence with zones of high predicted suitability, which indicates strong model performance in approximating the realized niche of the species. Low suitability is widespread across coastal lowlands, intensively cultivated plains, and arid areas of southeastern Türkiye, where habitat continuity remains limited and climatic constraints become more restrictive. Eastern Anatolia shows a similar pattern, especially at higher elevations under continental climatic conditions, where suitable areas occur only as scattered mountainous patches.

**Figure 2:**
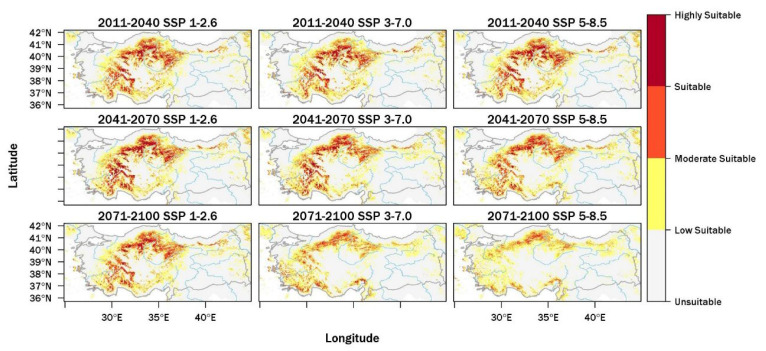
Average projected climate habitat suitability maps for the prickly juniper (*Juniperus oxycedrus* L.) under future climate scenarios in Türkiye. Projections are shown for three time periods (2011–2040, 2041–2070, and 2071–2100) and three Shared Socioeconomic Pathways (SSPs): optimistic (SSP 1-2.6), middle-of-the-road (SSP 3-7.0), and worst-case scenarios (SSP 5-8.5).

**Figure A2:**
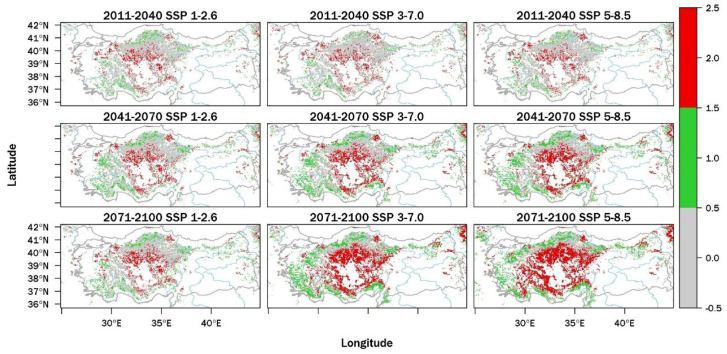
Average projected climate habitat suitability maps for the prickly juniper (*Juniperus oxycedrus* L.) under future climate scenarios in Türkiye under future climate scenarios. Projections are shown for three time periods (2011–2040, 2041–2070, and 2071–2100) and three Shared Socioeconomic Pathways (SSPs): optimistic (SSP 1-2.6), middle-of-the-road (SSP 3-7.0), and worst-case scenarios (SSP 5-8.5).

**Figure A3:**
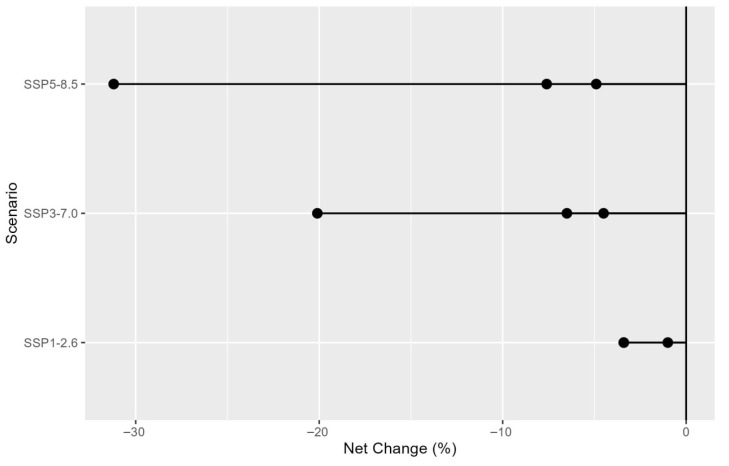
Projected habitat changes (%) for the prickly juniper (*Juniperus oxycedrus* L.) in Türkiye under future climate scenarios (SSP1-2.6, SSP3-7.0, and SSP5-8.5).

Model projections indicate substantial changes in the future habitat suitability of *J. oxycedrus* across Türkiye under all three Shared Socioeconomic Pathways and assessed time periods (Figure 2). In the near future (2011–2040), overall losses in suitable habitat remain limited, although clear differences between scenarios emerge, as shown in Appendix A, Figure A2. Under SSP1-2.6, suitable areas decrease by 6198 km^2^ (−3.4%), while larger reductions occur under SSP3-7.0 (−11,801 km^2^; −6.5%) and SSP5-8.5 (−8928 km^2^; −4.9%). During 2011–2040 under SSP1-2.6, areas of suitable habitat remain widely distributed across Central Anatolia and the interior Mediterranean region, with extensive suitability maintained across interior upland areas (Appendix A, Figure A3). Under SSP3-7.0 in the same period, reductions are mainly observed across plains and outer parts of the study area, whereas central and southern mountainous areas retain broad zones of suitability. Under SSP5-8.5, the spatial pattern remains similar, although suitable habitats become more fragmented toward the margins of Central Anatolia. During the mid-century period (2041–2070), habitat suitability declines more strongly under intermediate and high-emission scenarios. Under SSP1-2.6, losses remain small (−1817 km^2^; −1.0%), while reductions rise to −8153 km^2^ (−4.5%) under SSP3-7.0 and −13,735 km^2^ (−7.6%) under SSP5-8.5. For 2041–2070 under SSP1-2.6, suitable habitats continue to occupy large portions of Central Anatolia, although suitability decreases across lower-altitude areas and regions at the edges of the species’ current range. Under SSP3-7.0, suitable conditions retreat from plains and peripheral areas and remain mainly within elevated interior regions. Under SSP5-8.5, suitable habitats are more fragmented, and they mainly occur within the mountainous parts of Central Anatolia and the interior Mediterranean region.

The strongest reductions appear toward the end of the century (2071–2100). Under SSP1-2.6, the total extent of suitable habitats declines by 6083 km^2^ (−3.4%), whereas SSP3-7.0 results in a reduction of 36,252 km^2^ (−20.1%). The most extensive loss is projected under SSP5-8.5, where suitable habitats decline by 56,371 km^2^ (−31.2%). During the late-century period (2071–2100) under SSP1-2.6, suitable habitats remain across parts of Central Anatolia and the interior Mediterranean mountains. Overall habitat suitability is expected to decline in this period. Under SSP3-7.0, suitable areas are largely restricted to mountainous regions, while plateaus and low-altitude areas undergo substantial reductions. Under SSP5-8.5, suitable habitats are foreseen to remain only within mountain refugia. These areas occur mainly along the Taurus Mountains and in selected highland regions of Central and northern Türkiye. Across all scenarios and time periods, projections indicate a shift from broadly distributed habitat suitability toward increasingly restricted and fragmented patterns. Although *J. oxycedrus* tolerates a wide range of climatic and topographic conditions, its long-term occurrence appears closely linked to the extent and spatial arrangement of remaining suitable habitats. Late-century projections under high-emission scenarios indicate particularly high vulnerability across low-altitude regions and areas outside mountainous terrain, which emphasizes the importance of mountainous and interior upland regions as future strongholds for this species in Türkiye.

## 3. Discussion

### 3.1. Methodological Considerations and Limitations

ENM is widely used to evaluate potential species distributions and their responses to climate change by identifying climatically suitable areas based on occurrence records and environmental predictors. One of the key advantages of this approach is its ability to project future changes in habitat suitability under different climate scenarios, which provides valuable insights for conservation and restoration planning. In this study, the MaxEnt algorithm was selected due to its strong predictive performance with presence-only data and its reliability in modeling woody species distributions across heterogeneous landscapes. However, niche models primarily represent climatic suitability and do not incorporate all ecological processes. These limitations are discussed in more detail in the final part of this section. Despite these limitations, ENM remains an effective and widely accepted tool for identifying potential distribution limits and assessing climate-related changes in species distributions. The following sections present the ecological interpretation of the projected distribution patterns and their implications at both regional and broader scientific scales.

### 3.2. Interpretation of Projected Distribution Changes

The results of this study indicate that the distribution of *J. oxycedrus* in Central Anatolia is constrained by distinct ecological thresholds shaped jointly by climate and topography. The high discriminatory performance of the model (AUC = 0.869) and the statistically significant difference obtained in the null-model comparison indicate that the environmental niche of the species is not random but strongly structured by specific climatic and topographic limits. This pattern is consistent with the findings of previous studies, which showed that the distribution of woody species across the Mediterranean Basin is largely governed by climatic conditions [35,36].

Studies of species distribution modeling conducted in Mediterranean and semi-arid regions consistently demonstrate that temperature regimes and seasonal precipitation patterns play a key role in determining the potential range of woody taxa. In the Mediterranean Basin, Benito Garzón et al. [37] showed that intra-specific climatic responses are primarily governed by thermal gradients, drought intensity, and seasonal precipitation, which together represent dominant determinants of tree distributions. Ohlemüller et al. [36] further emphasized that species vulnerability under climate change depends not only on climatic suitability loss, but also on migration distances and accessibility to existing populations. In closed-basin systems such as Central Anatolia, where elevation gradients change sharply over short distances, the impacts of climate change are expected to be spatially heterogeneous. The distribution patterns identified here suggest that such heterogeneity will play a decisive role in shaping the future spatial organization of *J. oxycedrus* populations.

Given the current distribution conditions, the fact that dry season rainfall (bio14) is the most influential predictor clearly demonstrates that summer water stress is one of the key factors limiting this species’ distribution. This finding is supported by previous studies, which state that summer water limitations are a primary factor in the distribution of woody species under semi-arid and Mediterranean climates [38,39,40]. The strong contribution of precipitation during the driest month (bio14) indicates that *J. oxycedrus*, despite its drought tolerance, depends on a minimum level of moisture availability. Temperature seasonality (bio4) and annual mean temperature (bio1) show relevant contributions. These results demonstrate limited tolerance to thermal extremes and an affinity for climates with reduced seasonal extremes. Topographic variables, particularly slope, show the role of local site conditions in species distribution. In general, steeper terrain tends to have lower agricultural pressure and reduced intensive land use. Soil drainage and microclimatic conditions in these areas can be suitable as habitats for species. This interpretation is also consistent with studies from the Iberian Peninsula, which show that *J. oxycedrus* communities are concentrated in rocky, shallow-soiled and sloping areas [41,42]. Therefore, the distribution pattern in Central Anatolia reflects an ecological niche where topography shapes climatic effects at the local scale.

Current suitability maps indicate that high and moderate suitability areas are mainly concentrated in Central Anatolia, the interior Mediterranean region, and the leeward transition zones of the Black Sea Mountains, suggesting a strong dependence on mountainous and mid-elevation landscapes. In contrast, low suitability values dominate lowland plains, intensively cultivated areas, and the arid southeastern sector. This spatial differentiation indicates that climatic constraints combined with anthropogenic pressure further restrict the realized distribution of the species. The resulting discrepancy between potential and realized ranges is, therefore, likely to be largely driven by habitat fragmentation and land-use intensity. Similar spatial patterns have been reported for juniper communities throughout the Mediterranean Basin [42,43].

Future projections reveal that *J. oxycedrus* will only experience minor losses in the short and medium term but will experience significant reductions towards the end of the century, particularly under high-emission scenarios. The projected loss of 20–31% under SSP3-7.0 and SSP5-8.5 suggests that this species will likely adapt by moving into refugial landscapes rather than expanding its range. This pattern matches research indicating that woody species mainly respond to climate change by fragmenting and moving into refuge areas [36,37,44]. The concentration of suitable habitats in the Taurus Mountains and in mid- to high-elevation areas highlights the importance of topographic complexity for long-term persistence. Phylogeographic analyses have demonstrated that Juniper populations have a long history of persistence in Mediterranean refugia. This reflects the significant role of these landscapes in shaping their current genetic structure [45]. In a similar vein, patterns of genetic differentiation and morphological variation indicate that fragmented and peripheral populations often follow distinct evolutionary paths and contain locally adapted gene pools [46,47,48]. Although habitat fragmentation raises the risk of demographic isolation, peripheral populations remain important sources of genetic diversity and adaptive capacity under ongoing environmental change [49]. The contraction and fragmentation patterns projected for Central Anatolia, therefore, suggest that future genetic vulnerability will depend not only on isolation intensity but also on the capacity of refugial landscapes to maintain evolutionary resilience.

Land use history and human pressure form a critical background for interpreting these distribution dynamics. Historical forest losses in Central Anatolia have been found to be linked to anthropogenic activities [16,50,51]. The low suitability values observed in intensively cultivated and grazed landscapes indicate that climatic constraints act together with land use to shape the distribution of this species. Although abandoned lands may temporarily provide opportunities for juniper colonization, long-term habitat integrity is often compromised, as has been reported in Mediterranean ecosystems [43].

The ecological status of the Central Anatolian steppes and forest–steppe transition zones has long been debated. According to Kürschner and Parolly [3] and Tavşanoğlu and Bernardi [33], steppes historically coexisted as climate-adapted alternative biome states. These findings, in conjunction with the distribution patterns and edaphic constraints identified in this study, indicate that restoration and afforestation strategies must explicitly account for biome boundaries and microhabitat limitations.

Species vulnerability under climate change is shaped not only by losses in suitable areas, but also by migration distances and connectivity constraints [36]. Large-scale analyses show that fragmentation of suitable habitats constitutes a major limitation to long-term persistence [43]. The present results suggest that future suitable areas in Central Anatolia will become smaller, more isolated, and topographically constrained, highlighting the need to prioritize not only existing forest remnants but also the spatial continuity and transition corridors between them. Furthermore, the protection of peripheral and marginal populations, even at small population sizes and low genetic diversity, is crucial for maintaining species’ gene pools and ensuring the availability of genetic material for restoration efforts [49]. These conservation measures are essential for sustaining the long-term resilience of *J. oxycedrus* in the face of changing climatic conditions.

### 3.3. Relevance of the Findings and Implications for Future Research

Beyond the regional context, these findings contribute to a broader understanding of how forest distribution limits respond to climate change in semi-arid transition zones. The results show that the long-term presence of future forests will depend less on the overall extent of climatically suitable area and more on the spatial configuration, connectivity, and topographic complexity of remaining habitats. This emphasizes the crucial role of mountainous regions and transitional landscapes as long-term refuge areas. The use of an indicator species to identify these thresholds provides an effective way to understand forest distribution limits that can support climate adaptation planning and forest distribution assessment in other Mediterranean and semi-arid regions facing similar ecological constraints.

Finally, it is important to acknowledge the limitations of this study for future research. Although species distribution models primarily rely on climatic and topographic predictors, important drivers such as soil properties, land use patterns and intensity, disturbance regimes and demographic processes could not be explicitly incorporated into the modeling framework. Nevertheless, the robustness of the spatial patterns identified here is supported by consistent trends obtained across multiple GCMs, alternative SSP scenarios and different thresholding approaches. Future research integrating long-term monitoring data, process-based modeling and population genetic analyses is essential in order to refine these projections and better understand the mechanisms underlying range contraction, fragmentation and persistence. Such an integrative approach will provide a stronger basis for assessing population viability and designing effective conservation and restoration strategies in the context of ongoing climate change.

## 4. Materials and Methods

### 4.1. Study Area

**Figure 3:**
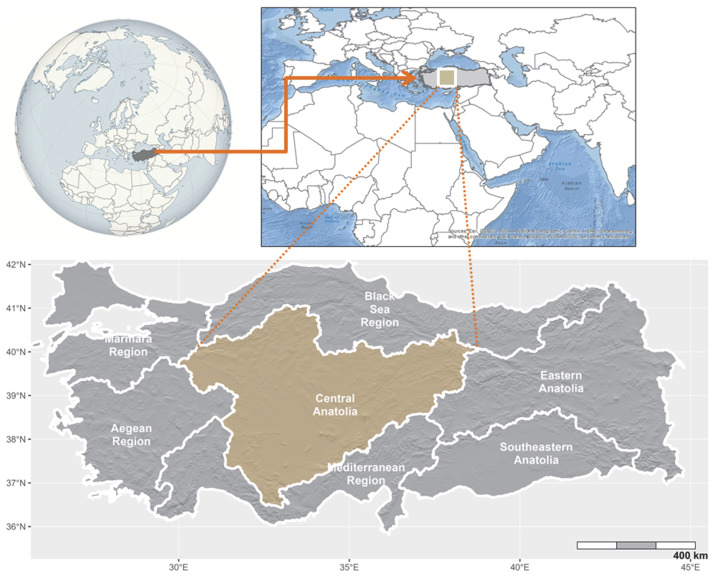
Geographical location of the study area.

The study area lies within the forest boundary that surrounds Central Anatolia, which represents the geographical center of Türkiye and covers approximately 150,000 km^2^. The landscape unit considered in this study corresponds to the area located between the forest belts surrounding Central Anatolia and covers nearly 110,000 km^2^ when lakes are excluded. Central Anatolia shows predominantly high-plateau morphology. Mountain systems such as Erciyes, the Sivrihisar Mountains, Elmadağ, Küre Boğazı, İdris, Çiçek, Dinek, Hasandağı, Melendiz Mountain, and the Karagüney Mountains represent important natural reserves or refugial areas within this landscape (Figure 3). Annual precipitation across the region ranges between 300 and 500 mm, and the mean annual temperature is approximately 10 °C. The climate of the study area is predominantly semi-arid [52]. The Karapınar district of Konya, which lies within the study area, represents the driest region in Türkiye. The drainage network of the study area remains sparse and includes two major river systems, the Sakarya and the Kızılırmak, with tributaries such as the Delice and Porsuk rivers. Saline wetlands occupy a substantial portion of the landscape, particularly Tuz Gölü, Sığla, Sultan Marshes, and the Ereğli Marshes. Reservoir lakes such as Sarıyar and Hirfanlı also cover extensive areas within the region.

Agricultural areas and rangelands dominate much of the study area. According to the Davis grid system, the region belongs to the Irano-Turanian vegetation zone [53]. The Taurus Mountains extend along the southern margin of the Konya Closed Basin, and cedar, black pine, and oak forests are present along their extensions and transition zones toward Central Anatolia. North of Tuz Gölü, particularly in areas that include Ankara, Eskişehir, Kırıkkale, and Kırşehir, the landscape exhibits more rugged high-plateau morphology [54]. Forest remnants remain widespread across the region, particularly in the form of open woodland formations. At different elevations, deciduous forests dominated by oak, mixed forests composed of oak, black pine, and juniper, and coniferous forests dominated by black pine occur [55]. Near the inner Black Sea forest boundary, which also defines the northern limit of the study area, remnant stands of Scots pine appear at higher elevations.

### 4.2. Target Species

*Juniperus oxycedrus* L. is a dioecious evergreen species that occurs as a shrub or a small tree. Most individuals reach heights of 3–4 m and display an open-branching structure with crowns that vary from conical and columnar to broadly round. In exposed settings, plants commonly have a habit of spreading low and remaining close to the ground. Under more favorable site conditions, the species assumes a tree form and reaches heights of up to 10 m. The bark has a gray-brown appearance and breaks into fine longitudinal fissures [56,57]. Young shoots show a rounded to slightly angular form, retain a green color when young, and later turn rust-brown. Leaves remain uniformly acicular, rigid, and are consistently arranged in whorls of three, with divergence angles of approximately 60–90° from the shoots. Leaf blades show a lanceolate outline, a lax posture, a pronounced keel, and an acuminate to mucronate apex. Dimensions range from (4–)10–20(–25) mm in length to 1.1–3 mm in width. The leaf surface appears green and displays two distinct whitish stomatal bands on the adaxial side [57,58]. Male and female reproductive organs occur singly within the leaf axils. Seed cones exhibit a berry-like structure and consist of three, occasionally up to six, fused scales. The fusion zone forms a characteristic three-rayed star. At maturity, cones show a nearly globose shape, reach diameters of 5–11 mm, and display orange, dark-red, purple, or bright-brown coloration with a distinctly rugose surface. Cone maturation requires two years and results in the presence of one to four seeds per cone [59]. The native range of the species covers the western and central Mediterranean basin and extends across temperate climatic zones [59,60]. The species has a natural distribution across a broad geographic area that includes Algeria, the Balearic Islands, Corsica, Cyprus, France, Iran, Italy, Morocco, Portugal, Sardinia, Spain, Syria, Tunisia, Türkiye, the Balkan Peninsula, and the Caucasus [60]. It commonly occurs across forest–steppe transition zones and is, therefore, considered an appropriate indicator species for assessing future changes in forest distribution limits.

### 4.3. Occurrence Data

Occurrence data for *J. oxycedrus* were obtained from the Global Biodiversity Information Facility (GBIF) [61] and from the authors’ field observations. All records passed through a data-cleaning procedure that removed duplicate entries, taxonomic errors, and records without sufficient supporting information. The dataset retained only confirmed occurrences supported by geographic coordinates or by accurate information suitable for spatial analysis. For Türkiye, regionally validated records supplemented the global dataset in order to improve representation of range–edge areas of the species. The final compilation consisted of 673 occurrence records, of which 333 originated from GBIF, and 340 originated from field observations. All occurrence data followed the WGS84 coordinate reference system, and positional accuracy received validation within ArcMap (v10.7; ESRI, Redlands, CA, USA). The combination of occurrence data derived from multiple field survey efforts across independent studies may improve model performance, yet such integration may also increase spatial autocorrelation [62]. To address sampling bias and to achieve a more even spatial distribution of records [63,64,65,66], a spatial thinning protocol was applied. This protocol preserved a single occurrence point within each 1 km radius grid cell. Record filtering followed implementation through the spThin R package [67]. This procedure reduced the dataset from 673 to 483 occurrence records. The study area corresponded to the known distribution of the species within Türkiye. The accessible area (M sensu Soberón & Peterson [68]) set the spatial extent of the analysis. The area covered latitudes between 35.7° N and 42.2° N and longitudes between 24.9° E and 44.9° E, corresponding to the territorial boundaries of Türkiye.

### 4.4. Environmental Data and Ecological Niche Modeling

Ecological niche modeling (ENM) followed the analytical framework presented in our previous study [29]. Species–environment relationships were quantified with the maximum entropy (MaxEnt) algorithm implemented in MaxEnt (version 3.4.1; Phillips et al. [69,70]), which served to estimate habitat suitability under both current and future climatic conditions. Model evaluation followed a 10-fold cross-validation scheme, and the occurrence data were randomly assigned to training and testing subsets. Model complexity and predictive performance were assessed and optimized with the ENMeval package (v2.0.5), which ensured an explicit balance between model fit and predictive capacity [71,72]. The parameters for the models included the testing of the regularization multiplier, starting from 0.5 to 4, in intervals of 0.5, for the six feature classes: l, lq, h, lqh, lqhp, and lqhpt. More details regarding the approach can be obtained from Özcan et al. [29]. Before model construction, environmental predictors were screened for multicollinearity. Variables showing variance inflation factor (VIF) values greater than 5 were excluded. This exclusion was based on a correlation threshold of 0.7, as recommended in ENM studies [73,74,75]. Following this procedure and considering the ecological preferences of the species, seven predictors were retained: annual mean temperature (bio1), isothermality (bio3), temperature seasonality (bio4), precipitation of the wettest month (bio13), precipitation of the driest month (bio14), slope, and topographic position index (TPI). Model predictions produced as continuous cloglog probabilities were subsequently converted into binary presence–absence maps. To limit bias related to over- or under-prediction, multiple threshold-selection approaches were applied in line with Hellegers et al. [76], including maximum test sensitivity and specificity, equal training sensitivity and specificity, 10th percentile training presence combined with balanced training omission, predicted area and threshold, and balanced training omission, predicted area and threshold. Outputs from all thresholding approaches were combined into a single map with values from 0 to 4. Values denote the number of methods that support presence at each pixel. Presence–absence classification was based on a consensus threshold of ≥2, while a higher threshold of ≥3 was used for sensitivity analysis. All remaining methodological steps, including model calibration and evaluation, climate projections under multiple Shared Socioeconomic Pathways, spatial resampling procedures, and area calculations, were conducted as described in the referenced study and are, therefore, not repeated here.

## 5. Conclusions

The study provides strong and quantitative evidence that the boundaries of the forest–steppe in Central Anatolia are controlled by distinct ecological thresholds shaped jointly by climate and topography. Our results suggest that suitable habitats for *J. oxycedrus* are projected to decrease in extent under future climate scenarios and are expected to become more fragmented and increasingly restricted to topographically complex landscapes. The significant impact of dry season rainfall, temperature seasonality and slope demonstrates that forest distribution limits are strongly constrained by specific climatic and topographic conditions. Future projections indicate that mountainous and mid- to high-elevation areas are likely to remain the primary locations of climatically suitable habitats.

By identifying the climatic and topographic factors that define forest distribution limits, this study provides spatially explicit insights into how forest boundaries may respond to climate change in semi-arid transition zones. These findings contribute to a broader understanding of forest distribution responses to climate change, particularly in Mediterranean regions where similar environmental constraints exist. However, it is important to emphasize that the model does not incorporate several important factors, such as land use, soil properties, disturbance regimes, and species interactions, which may also influence the realized distribution of the species. Therefore, the results should be interpreted as projections of climatic suitability rather than exact predictions of future species distribution.

## Data Availability

The data presented in this study are available on request from the corresponding author.

## Abbreviations

The following abbreviations are used in this manuscript:

ENM	Ecological niche modeling
MAXENT	Maximum entropy
AUC	Area under curve
TPI	Topographic position index

## Appendix A

**Table A1:** Summary statistics of MaxEnt model performance and significance tests, including training and validation metrics (auc.train: Area Under the Curve; cbi.train: Continuous Boyce Index; auc.val: validation AUC; auc.diff: AUC difference; cbi.val: validation Continuous Boyce Index; or.mtp: minimum training presence omission rate; or.10p: 10th percentile omission rate; emp.mean: empirical mean; emp.sd: empirical standard deviation; null.mean: null mean; null.sd: null standard deviation; zscore: z-score; pvalue: *p*-value).

	Statistic	auc.train	cbi.train	auc.val	auc.diff	cbi.val	or.mtp	or.10p
1	emp.mean	0.8958275	0.995	0.88183375	0.022921898	0.9156	0.00625	0.120833333
2	emp.sd	NA	NA	0.02333767	0.017221619	0.038750484	0.01406143	0.036483126
3	null.mean	0.592712576	0.94624	0.634242925	0.085019452	0.360306	0.00625	0.074416667
4	null.sd	0.010712951	0.058450552	0.012514879	0.010023558	0.092611039	0.0058402	0.0158573
5	zscore	28.29425001	0.834209396	19.78371744	−6.195160537	5.995980683	0	2.927148094
6	pvalue	2.0337 × 10^−176^	0.202081499	2.05614 × 10^−87^	2.91128 × 10^−10^	1.01131 × 10^−09^	0.5	0.99828957

**Table A2:** Summary statistics and model selection criteria for candidate MaxEnt models across different feature class combinations and regularization multiplier settings (fc: feature class; rm: regularization multiplier; tune.args: tuning arguments; auc.train: Area Under the Curve; cbi.train: Continuous Boyce Index; auc.diff.avg: mean AUC difference; auc.diff.sd: standard deviation of AUC difference; auc.val.avg: validation AUC; auc.val.sd: standard deviation of validation AUC; cbi.val.avg: validation Continuous Boyce Index; cbi.val.sd: standard deviation of validation Continuous Boyce Index; or.10p.avg: 10th percentile omission rate; or.10p.sd: standard deviation of 10th percentile omission rate; or.mtp.avg: minimum training presence omission rate; or.mtp.sd: standard deviation of minimum training presence omission rate; delta.AICc: difference in corrected Akaike Information Criterion; AICc: corrected Akaike Information Criterion; w.AIC: Akaike weight; ncoef: number of model coefficients).

	fc	rm	tune.args	auc. train	cbi.train	auc.diff. avg	auc.diff.sd	auc.val. avg	auc.val. sd	cbi.val. avg	cbi.val. sd	or.10p. avg	or.10p. sd	or.mtp.avg	or.mtp. sd	AICc	delta.AICc	w.AIC	ncoef
1	L	0.5	fc.L_rm.0.5	0.783	0.885	0.027	0.023	0.780	0.032	0.852	0.065	0.106	0.050	0.002	0.007	13,200.15	840.10	3.7 × 10^−183^	7
2	LQ	0.5	fc.LQ_rm.0.5	0.835	0.998	0.023	0.022	0.831	0.029	0.918	0.049	0.102	0.033	0.002	0.007	12,925.97	565.92	1.3 × 10^−123^	13
3	H	0.5	fc.H_rm.0.5	0.891	0.988	0.021	0.018	0.879	0.023	0.901	0.040	0.121	0.035	0.004	0.013	12,381.6	21.55	2.1 × 10^−05^	56
4	LQH	0.5	fc.LQH_rm.0.5	0.891	0.988	0.021	0.018	0.879	0.023	0.900	0.040	0.121	0.035	0.004	0.013	12,379.01	18.95	7.7 × 10^−05^	55
5	LQHP	0.5	fc.LQHP_rm.0.5	0.896	0.995	0.023	0.017	0.882	0.023	0.916	0.039	0.121	0.036	0.006	0.014	12,360.05	0.00	1.0 × 10^00^	59
6	LQHPT	0.5	fc.LQHPT_rm.0.5	0.905	0.991	0.030	0.020	0.879	0.022	0.889	0.043	0.156	0.046	0.008	0.015	12,417.1	57.04	4.1 × 10^−13^	105
7	L	1	fc.L_rm.1	0.783	0.884	0.027	0.023	0.780	0.032	0.845	0.066	0.106	0.050	0.002	0.007	13,200.94	840.89	2.5 × 10^−183^	7
8	LQ	1	fc.LQ_rm.1	0.827	0.994	0.023	0.023	0.824	0.030	0.934	0.029	0.104	0.039	0.002	0.007	12,965.04	604.99	4.3 × 10^−132^	11
9	H	1	fc.H_rm.1	0.888	0.991	0.020	0.019	0.878	0.024	0.897	0.053	0.121	0.036	0.002	0.007	12,400.5	40.45	1.6 × 10^−09^	41
10	LQH	1	fc.LQH_rm.1	0.888	0.991	0.020	0.019	0.878	0.024	0.894	0.056	0.119	0.038	0.002	0.007	12,400.41	40.36	1.7 × 10^−09^	41
11	LQHP	1	fc.LQHP_rm.1	0.890	0.993	0.021	0.019	0.879	0.025	0.907	0.041	0.115	0.037	0.004	0.009	12,390.91	30.86	2.0 × 10^−07^	43
12	LQHPT	1	fc.LQHPT_rm.1	0.893	0.986	0.022	0.019	0.878	0.022	0.864	0.048	0.131	0.035	0.004	0.013	12,394.11	34.06	4.0 × 10^−08^	65
13	L	1.5	fc.L_rm.1.5	0.783	0.875	0.027	0.023	0.780	0.033	0.839	0.068	0.106	0.050	0.002	0.007	13,202.05	842.00	1.5 × 10^−183^	7
14	LQ	1.5	fc.LQ_rm.1.5	0.823	0.989	0.026	0.023	0.819	0.033	0.916	0.028	0.108	0.049	0.002	0.007	12,987.98	627.93	4.4 × 10^−137^	10
15	H	1.5	fc.H_rm.1.5	0.885	0.974	0.019	0.018	0.877	0.023	0.874	0.047	0.113	0.043	0.002	0.007	12,430.76	70.70	4.4 × 10^−16^	33
16	LQH	1.5	fc.LQH_rm.1.5	0.885	0.973	0.019	0.018	0.876	0.023	0.874	0.045	0.113	0.043	0.002	0.007	12,430.73	70.68	4.5 × 10^−16^	33
17	LQHP	1.5	fc.LQHP_rm.1.5	0.885	0.975	0.020	0.019	0.876	0.024	0.874	0.060	0.119	0.038	0.004	0.009	12,428.86	68.81	1.1 × 10^−15^	35
18	LQHPT	1.5	fc.LQHPT_rm.1.5	0.888	0.979	0.019	0.018	0.876	0.021	0.864	0.056	0.125	0.033	0.004	0.009	12,400.94	40.89	1.3 × 10^−09^	45
19	L	2	fc.L_rm.2	0.783	0.876	0.027	0.023	0.780	0.033	0.836	0.068	0.104	0.049	0.002	0.007	13,203.58	843.53	6.8 × 10^−184^	7
20	LQ	2	fc.LQ_rm.2	0.817	0.974	0.031	0.022	0.812	0.036	0.898	0.048	0.106	0.041	0.004	0.013	13,026.47	666.41	2.0 × 10^−145^	10
21	H	2	fc.H_rm.2	0.882	0.974	0.019	0.018	0.875	0.023	0.877	0.037	0.119	0.038	0.004	0.009	12,461.06	101.01	1.2 × 10^−22^	26
22	LQH	2	fc.LQH_rm.2	0.882	0.972	0.019	0.018	0.875	0.023	0.874	0.046	0.119	0.038	0.004	0.009	12,461.06	101.00	1.2 × 10^−22^	26
23	LQHP	2	fc.LQHP_rm.2	0.883	0.977	0.019	0.019	0.874	0.024	0.871	0.058	0.123	0.037	0.004	0.009	12,464.79	104.74	1.8 × 10^−23^	30
24	LQHPT	2	fc.LQHPT_rm.2	0.885	0.978	0.018	0.017	0.875	0.021	0.877	0.036	0.125	0.037	0.004	0.009	12,421.35	61.30	4.9 × 10^−14^	34
25	L	2.5	fc.L_rm.2.5	0.783	0.863	0.027	0.024	0.780	0.033	0.829	0.072	0.104	0.049	0.002	0.007	13,205.49	845.44	2.6 × 10^−184^	7
26	LQ	2.5	fc.LQ_rm.2.5	0.812	0.933	0.032	0.022	0.810	0.037	0.896	0.053	0.100	0.052	0.004	0.013	13,056.7	696.65	5.3 × 10^−152^	8
27	H	2.5	fc.H_rm.2.5	0.880	0.970	0.019	0.018	0.873	0.023	0.878	0.029	0.119	0.050	0.004	0.009	12,498.95	138.90	6.9 × 10^−31^	24
28	LQH	2.5	fc.LQH_rm.2.5	0.880	0.969	0.018	0.018	0.873	0.023	0.872	0.034	0.121	0.048	0.004	0.009	12,498.98	138.93	6.8 × 10^−31^	24
29	LQHP	2.5	fc.LQHP_rm.2.5	0.880	0.981	0.019	0.019	0.872	0.024	0.860	0.067	0.121	0.049	0.004	0.009	12,496.78	136.73	2.0 × 10^−30^	25
30	LQHPT	2.5	fc.LQHPT_rm.2.5	0.883	0.969	0.018	0.016	0.874	0.021	0.867	0.043	0.121	0.040	0.004	0.009	1,2444.81	84.76	3.9 × 10^−19^	27
31	L	3	fc.L_rm.3	0.783	0.860	0.027	0.024	0.780	0.033	0.823	0.074	0.104	0.049	0.002	0.007	13,207.74	847.69	8.5 × 10^−185^	7
32	LQ	3	fc.LQ_rm.3	0.810	0.911	0.032	0.023	0.808	0.036	0.892	0.056	0.100	0.052	0.004	0.013	13,075.33	715.28	4.8 × 10^−156^	9
33	H	3	fc.H_rm.3	0.878	0.973	0.018	0.018	0.872	0.024	0.881	0.036	0.121	0.047	0.004	0.009	12,527.53	167.48	4.3 × 10^−37^	20
34	LQH	3	fc.LQH_rm.3	0.878	0.974	0.018	0.018	0.871	0.023	0.882	0.029	0.121	0.046	0.004	0.009	12,529.96	169.90	1.3 × 10^−37^	21
35	LQHP	3	fc.LQHP_rm.3	0.878	0.983	0.019	0.019	0.870	0.024	0.891	0.024	0.115	0.050	0.004	0.009	12,532.25	172.20	4.0 × 10^−38^	24
36	LQHPT	3	fc.LQHPT_rm.3	0.881	0.975	0.018	0.016	0.872	0.021	0.866	0.040	0.117	0.051	0.004	0.009	12,474.87	114.82	1.2 × 10^−25^	25
37	L	3.5	fc.L_rm.3.5	0.783	0.840	0.028	0.024	0.780	0.034	0.818	0.078	0.104	0.049	0.002	0.007	13,210.34	850.29	2.3 × 10^−185^	7
38	LQ	3.5	fc.LQ_rm.3.5	0.807	0.897	0.032	0.023	0.805	0.036	0.883	0.064	0.100	0.052	0.004	0.013	13,093.69	733.63	4.9 × 10^−160^	9
39	H	3.5	fc.H_rm.3.5	0.876	0.968	0.018	0.018	0.871	0.023	0.867	0.054	0.119	0.042	0.002	0.007	12,554.52	194.47	5.9 × 10^−43^	19
40	LQH	3.5	fc.LQH_rm.3.5	0.876	0.966	0.018	0.018	0.870	0.023	0.878	0.042	0.119	0.044	0.002	0.007	12,554.93	194.88	4.8 × 10^−43^	19
41	LQHP	3.5	fc.LQHP_rm.3.5	0.876	0.976	0.019	0.019	0.869	0.024	0.894	0.028	0.108	0.050	0.004	0.009	12,555.1	195.05	4.4 × 10^−43^	20
42	LQHPT	3.5	fc.LQHPT_rm.3.5	0.879	0.985	0.018	0.016	0.871	0.021	0.853	0.041	0.113	0.045	0.004	0.009	12,506.28	146.23	1.8 × 10^−32^	23
43	L	4	fc.L_rm.4	0.783	0.834	0.028	0.025	0.780	0.034	0.808	0.087	0.102	0.051	0.002	0.007	13,213.27	853.22	5.3 × 10^−186^	7
44	LQ	4	fc.LQ_rm.4	0.805	0.880	0.032	0.023	0.803	0.036	0.869	0.079	0.102	0.049	0.004	0.013	13,106.94	746.88	6.6 × 10^−163^	8
45	H	4	fc.H_rm.4	0.875	0.971	0.018	0.018	0.869	0.023	0.856	0.076	0.117	0.048	0.002	0.007	12,582.94	222.89	4.0 × 10^−49^	18
46	LQH	4	fc.LQH_rm.4	0.875	0.972	0.018	0.018	0.869	0.023	0.870	0.053	0.113	0.045	0.002	0.007	12,587.05	227.00	5.1 × 10^−50^	20
47	LQHP	4	fc.LQHP_rm.4	0.874	0.976	0.019	0.019	0.868	0.024	0.887	0.034	0.110	0.047	0.004	0.009	12,588.83	228.77	2.1 × 10^−50^	21
48	LQHPT	4	fc.LQHPT_rm.4	0.877	0.989	0.018	0.016	0.869	0.021	0.857	0.043	0.108	0.044	0.004	0.009	12,533.97	173.92	1.7 × 10^−38^	21
